# DNA Methylation Episignatures in Neurodevelopmental Disorders Associated with Large Structural Copy Number Variants: Clinical Implications

**DOI:** 10.3390/ijms23147862

**Published:** 2022-07-16

**Authors:** Kathleen Rooney, Bekim Sadikovic

**Affiliations:** 1 Department of Pathology and Laboratory Medicine, Western University, London, ON N6A 3K7, Canada; kathleen.rooney@lhsc.on.ca; 2 Verspeeten Clinical Genome Centre, London Health Sciences Centre, London, ON N6A 5W9, Canada

**Keywords:** DNA methylation, episignature, epigenetics, copy number variant, neurodevelopmental disorder, genomic disorder

## Abstract

Large structural chromosomal deletions and duplications, referred to as copy number variants (CNVs), play a role in the pathogenesis of neurodevelopmental disorders (NDDs) through effects on gene dosage. This review focuses on our current understanding of genomic disorders that arise from large structural chromosome rearrangements in patients with NDDs, as well as difficulties in overlap of clinical presentation and molecular diagnosis. We discuss the implications of epigenetics, specifically DNA methylation (DNAm), in NDDs and genomic disorders, and consider the implications and clinical impact of copy number and genomic DNAm testing in patients with suspected genetic NDDs. We summarize evidence of global methylation episignatures in CNV-associated disorders that can be used in the diagnostic pathway and may provide insights into the molecular pathogenesis of genomic disorders. Finally, we discuss the potential for combining CNV and DNAm assessment into a single diagnostic assay.

## 1. Introduction

Neurodevelopmental disorders (NDDs) are a class of neurological and neuropsychiatric conditions that manifest in childhood during the developmental phase and persist throughout life [1]. They affect development of the central nervous system and can lead to brain dysfunction, resulting in limitations or impairment in cognition, motor performance, vision, hearing, speech and behavior [2]. NDDs include, but are not limited to, autistic spectrum disorder (ASD), intellectual disability (ID), attention deficit hyperactivity disorder (ADHD) and epilepsy, all of which show high rates of comorbidity and phenotypic overlap [3]. They are estimated to affect approximately 3% of children worldwide [4,5] and therefore, collectively represent a significant impact to families and health care systems.

NDDs present with a broad range of genetic and phenotypic heterogeneity, and clinical presentations are often non-specific. Genetics plays an important role in the etiology of hereditary NDDs. Genetic mutations associated with NDDs vary in size from single nucleotide variants (SNVs) to whole chromosome aneuploidies [6]. Due to the phenotypic overlap exhibited, in addition to targeted gene sequencing approaches, genetic testing often involves global genomic screening including chromosomal microarray analysis (CMA), exome or whole genome sequencing (WES, WGS), or classically G-banded chromosome karyotyping (karyotyping). CMA, considered the first-tier diagnostic test for patients with NDDs, has been used clinically for nearly two decades [7,8] to detect structural imbalances involving deletion or duplication of genetic material, collectively termed copy number variants (CNVs). Whilst some of the first CMAs included bacterial artificial chromosome (BAC) clones in an array-based comparative genomic hybridization (aCGH) with genome-wide coverage at approximately 1 Mb intervals [9,10], more recent platforms involving oligonucleotide or high-resolution single nucleotide polymorphism (SNP) arrays may reach a resolution of a few hundred base pairs [11,12]. Therefore, genomic imbalances beyond the resolution of karyotyping (minimum detection sizes 3–7 Mb) [13,14] are now routinely detected. SNP arrays offer the highest resolution of commercially available microarrays and are designed to determine genotype, structural imbalances, genomic aneuploidy, and loss of heterozygosity [15].

Microarray platforms can be customized to increase coverage and resolution in clinically relevant regions and regions associated with well-defined genomic syndromes. In addition to probe-coverage enriched regions, clinical use microarray platforms have probes equally spaced across the rest of the genome, termed “backbone” coverage. The combination of high probe densities and optimized targeted design is aimed at reducing the rate of ambiguous findings termed variants of uncertain significance (VUSs), since, at this time, there is a limited understanding of the impact of CNVs outside of protein coding regions.

## 2. The Role of CNVs in Genomic Disorders

Structural variants, defined as “alterations that involve segments of DNA larger than 1 kb” [13], include CNVs linked to phenotypic variation and disease susceptibility [16]. CNVs can contain millions of nucleotides, multiple genes and regulatory elements. Tuzun et al. suggested that individuals carry on average 250 CNVs [17]. As such, compared with SNVs, CNVs are reported to be responsible for more than ten times the total heritable sequence differences observed in the general population [18]. CNVs are also described as polymorphisms in association with several non-pathological conditions, e.g., those involved in variation in olfactory perception [19]. The presence of CNVs in non-pathological conditions can present challenges in the interpretation and classification of variants, especially in the absence of functional studies.

Genomic disorders are a group of genetic conditions caused by CNVs affecting dosage sensitive genes or genes critical for normal development or maintenance and/or their regulatory elements [20]. Recurrent disorders, those with common start and stop breakpoints, include CNVs that are similar in size and gene content, and typically present with similar phenotypes, e.g., deletions and duplications of 17p11.2 (Smith–Magenis and Potocki–Lupski syndrome), 7q11.23 (Williams syndrome), 15q11.2 (Prader–Willi/Angelman syndrome) and 17q21.31 (Koolen–de Vries syndrome) [6,21,22]. In contrast, non-recurrent disorders show variability in size and gene content (typically there is a common region of overlap). Phenotypes in these patients vary substantially, e.g., deletions of 22q13.3 in Phelan–McDermid syndrome (PHMDS) or deletions of 9q34.3 in Kleefstra syndrome [6].

Segmental duplications (also known as low copy repeats; LCRs) are blocks of DNA ranging from 1–400 kb that occur throughout the genome and typically share a high level (>90%) of sequence identity [23,24]. Many structural rearrangements, including CNVs, are mediated by LCRs through non-allelic homologous recombination (NAHR) [25]. These LCRs are highly prone to rearrangements which can result in genomic imbalances, including those associated with the common CNV-related disorders. 

A recent study assessed the prevalence and inheritance of CNVs associated with NDDs and estimated recurrent CNVs present in approximately 1 in 200 live births [26]. These results indicate that while individual CNVs may be rare, collectively they contribute significantly to NDDs. The most common CNVs observed in NDDs are those associated with genomic disorders [6], and a recent study estimated deletions of the 16p11.2 proximal region, 17q12, and 1q21.1 regions, and duplications of 15q11.2, 22q11.2, and the 16p11.2 distal region as the most common [26]. These findings vary from previous studies where duplications of 2q13 and deletions of 22q11.2, 15q11.2 and 1p36 were among the most common [21]. These differences likely reflect increased resolution of microarray platforms, as well as a decrease in ascertainment bias. 

Considerable research to date has focused on genes within the CNV regions of genomic disorders and how dosage sensitivity may be responsible for the observed phenotypes. While this research has identified some causative or candidate genes for specific Mendelian disorders and phenotypes, the genetic contribution for the majority of the observed clinical phenotypes in CNV disorders are not well defined [21]. One example of a disorder where a contributory gene has been identified is the 5q35 deletion. 5q35 deletion is predominantly mediated by NAHR and is associated with Sotos syndrome 1, where haploinsufficiency of the nuclear receptor-binding set domain protein (*NSD1)* gene contained within this region is shown, on its own, to be causative for Sotos [27]. This is similar to findings in Smith–Magenis syndrome, where deletions of 17p11.2 are responsible for 90% of causative variants, while approximately 5% are the result of point mutations in the retinoic acid-induced 1 (*RAI1*) gene contained within this region [22,23,24,25,26,27,28]. This is in contrast to disorders such as 16p11.2 and 22q11.2 deletion and duplication syndromes where no single candidate gene has been identified.

Overall, CNVs may contribute to the clinical features observed in genomic disorders through dosage sensitivity, via haploinsufficiency (as described for Sotos and Smith–Magenis), triplosensitivity (e.g., 22q11.2 duplication syndrome) or imprinting effects (Prader-Willi and Angelman syndromes), or through disruption of gene expression via positional effects, including disruption of transcriptional regulatory elements and changes in the chromatin structure.

## 3. Clinical Identification of CNVs in Patients with NDDs

CMA screening for CNVs in patients with NDDs has an estimated diagnostic yield of approximately 15–20% [21,22,23,24,25,26,27,28,29], which is a significant increase from karyotyping (3%) [7]. Newer molecular techniques such as WES or WGS in patients with developmental delay (DD) or ID have a reported diagnostic yield of approximately 25–36% [30,31,32,33]. Although these technological advancements have improved diagnostic capabilities in these disorders, half to two thirds of patients with suspected genetic conditions remain without a diagnosis [32,33,34]. 

This ‘diagnostic odyssey’, the time from initial consultation to diagnosis, often involves multiple clinical evaluations and laboratory tests spanning years [35,36], resulting in significant social and economic burden on both families and health care systems. In addition to CMA as the first-tier screen [7], in males with DD it is often accompanied by assessment for Fragile-X syndrome (FRX). FRX is an X-linked dominant condition and the most common inherited cause of ID. FRX results from abnormal expansion of the CGG trinucleotide repeat (>200 repeats) located in the promoter of the fragile X messenger ribonucleoprotein 1 (*FMR1)* gene, resulting in promotor DNA hypermethylation and gene silencing [37]. FRX can also be the result of deletions of Xq27.3 containing the *FMR1* gene [38]. Reflexive genetic testing, whereby the results of previous tests are used to guide further investigations, include DNA methylation (DNAm) analysis in individuals with CNVs at common imprinting loci, e.g., 15q11.1 and 11p15.5 regions. The average time to diagnosis in patients referred for genetic testing is estimated at 1–8 years [7,35,36,37,38,39], and the cost to healthcare is often difficult to estimate or missing from research. Recent studies describe the substantial positive medical and psychosocial outcome of receiving a genetic diagnosis [40,41]. Therefore, development of novel diagnostic technologies or testing strategies to shorten the diagnostic odyssey or increase the diagnostic yield represent an ongoing priority in NDD research [42]. 

Many recent studies have demonstrated disruption of genomic DNAm as a functional consequence of genetic defects in patients with NDDs [43,44,45,46]. There is emerging evidence of similar DNAm disruptions as epigenetic biomarkers for CNV disorders and their associated clinical phenotypes.

## 4. The Role of Epigenetics in NDDs and Subsequent Episignature Mapping

Epigenetics refers to mitotically heritable gene regulatory mechanisms without changes in the DNA sequence [47,48]. Epigenetic regulation of gene expression occurs at the level of chromatin and typically involves processes that modify chromatin or histones, the proteins around which DNA is wrapped, or covalent modifications in the associated DNA molecule [49]. DNAm is the most extensively studied epigenetic modification and refers to the mechanism of addition or removal of a methyl group to cytosine nucleotides [50]. Most cytosines subject to DNAm are adjacent to guanine residues and referred to as CpG dinucleotides (CpGs) [50]. High density clusters of CpGs, often associated with gene promoters, are referred to as CpG islands [50]. Unmethylated (hypomethylated) CpGs and CpG islands are generally associated with open, transcriptionally accessible chromatin, while DNA hypermethylation correlates with compact, transcriptionally repressive chromatin [51]. The majority of CpGs in the human genome are methylated except for those contained within CpG islands [52]. Therefore, in addition to affecting chromatin states and stability, disruptions in DNAm patterns can alter gene expression [51]. 

An increasing number of chromatin and epigenetic regulatory genes are becoming implicated in a variety of NDDs. Mutations in these genes result in DNAm episignatures, whole genome methylation changes, which are routinely detectable in the peripheral blood of patients affected by these disorders [43]. An episignature is defined as a recurring epigenetic pattern associated with a common genetic or environmental etiology in a disorder-specific patient population. Episignatures are highly sensitive and specific biomarkers that can be used to help resolve ambiguous clinical and genetic findings, and for screening patients with suspected genetic conditions [45]. Episignatures have the potential to provide insight into functional effects of certain mutations and genomic alterations on widespread DNAm and their contribution to the pathophysiology of genetic disorders [46].

Histone modifications refer to the chemical modification of histone tails by processes including methylation, acetylation, phosphorylation and ubiquitination. Histone tails are loosely structured protein segments that can mediate interaction between nucleosomes, and their modifications can result in either condensed or more relaxed chromatin, ultimately exhibiting an effect on gene transcription, as well as accessibility of DNA to other chromatin remodeling factors, including those involved in DNA methylation. Our group and others have identified unique episignatures in multiple NDDs that are the consequence of mutations in genes associated with histone modification [44], including, e.g., Kabuki syndrome caused by mutations in the lysine-specific methyltransferase 2D gene (*KMT2D*) [53]. We have mapped episignatures in several other histone modifying genes including lysine-specific methyltransferase 2B (*KMT2B*), set domain-containing protein 2 (*SETD2*), creb-binding protein (*CREBBP*), lysine acetyltransferase 6A (*KAT6A*) and lysine demethylase 4B (*KDM4B*) [43]. In addition, unique episignatures have also been reported in genes associated with the removal of histone methylation marks, the so-called “eraser” genes, such as the histone lysine demethylase 5C gene (*KDM5C*) in Claes–Jensen syndrome [54]. Histone modifications work in concert with DNAm to affect chromatin remodeling and gene expression.

The DNAm reaction is catalyzed by enzymes known as DNA methyltransferases (DNMT), which are responsible for mediating the transfer of the methyl group from S-adenosylmethionine (SAM) to cytosine residues. Robust episignatures have been reported in NDDs caused by mutations in the DNA methyltransferase genes *DNMT1*, *DNMT3A* and *DNMT3B* [44,55]. These genes are involved in the establishment and maintenance of DNAm during DNA replication and are termed “writers” since they are responsible for the addition of the methyl group to cytosines. Unique episignatures in two disorders are associated with mutations in *DNMT1*, hereditary sensory neuropathy with dementia and hearing loss (HSNDHL), and autosomal dominant cerebellar ataxia, deafness and narcolepsy (ADCADN) ([56,57]), while loss of function mutations in *DNMT3A* result in an episignature in Tatton–Brown–Rahman syndrome (TBRS) [44,58]. Mutations in *DNMT3B*, which cause immunodeficiency, centromere instability and facial anomalies (ICF) syndrome, also result in genomic defects in DNAm [59]. We recently demonstrated a genome-wide DNA hypermethylation episignature in a DNA demethylation gene Tet methylcytosine dioxygenase 3 (*TET3*), an “eraser” gene that opposes the writer function of *DNMT1* [60]. Mutations in the highly conserved catalytic domain of TET3 cause Beck–Fahrner syndrome (BEFAHRS). Inheritance patterns of BEFAHRS vary and include autosomal dominant or recessive forms [61]. Through episignature mapping, we were able to differentiate between affected individuals with mono- and bi-allelic mutations [60].

To date, chromatin remodeling genes comprise the largest group of epigenetic modifier genes with mapped episignatures, e.g., truncating mutations in the SNF2-related CBP activator protein gene (*SRCAP*) result in an episignature specific for Floating-Harbor syndrome [62]. Schenkel et al. reported a unique methylation profile associated with mutations in the ATRX chromatin remodeler gene *ATRX* in alpha-thalassemia X-linked intellectual disability syndrome [63]. In addition, our group previously described a shared DNAm episignature in Coffrin–Siris and Nicolaides–Baraitser syndromes (NCBRS) [64], which are two phenotypically similar NDDs associated with mutations in subunits of the BAF chromatin remodeling complex (commonly referred to as BAFopathies). This study described a shared BAFopathies episignature and supported the findings from previous studies suggesting that these conditions represent a disease spectrum rather than two distinct disorders [65]. Furthermore, this study indicates that methylation analysis may uncover or provide further support for the “relatedness” of genes and their disorders. In a subsequent study by our group, we described a new syndrome involving the BAF complex and the SWI/SNF-related matrix-associated, actin-dependent regulator of the chromatin gene (*SMARCA2*)—a gene reported in multiple individuals with NCBRS. This new syndrome was identified based on unique methylation patterns observed in individuals with intragenic variants located in the helicase domain of the *SMARCA2* gene compared to individuals with pathogenic variants located outside the helicase domain [66]. In support of these findings, clinical features of patients with *SMARCA2* helicase domain mutations exhibited a common phenotype distinct from NCBRS. Similarly, functional studies in yeast supported a different molecular mechanism underlying these two disorders [66]. Therefore, by analyzing variants from multiple regions within a gene, we were able to identify two unique episignatures and uncover functional data to explain the phenotypic differences seen between patients harboring variants in the same gene, resulting in the discovery of a new syndrome.

Interestingly, two distinct domain-specific episignatures have also been described in Helsmoortel-van der Aa syndrome associated with dominant negative truncating mutations in the activity-dependent neuroprotector homeobox gene (*ADNP*), which has chromatin regulatory functions [67]. These signatures were partially opposing, with mutations in the N- and C-terminus resulting in a predominantly hypomethylated signature; in contrast, mutations centered on the nuclear localization sequence resulted in a predominant hypermethylation signature. A subsequent study confirmed phenotypic differences between patients that correlated with the two episignatures [68].

Genes whose primary function is not associated with epigenetic and chromatin regulatory mechanisms, such as ubiquitin-conjugating enzyme E2 A (*UBE2A*) and spermine synthase (*SMS*) in X-linked syndromic forms of mental retardation—Nascimento and Snyder–Robinson types, respectively [44]—have also shown evidence of unique episignatures. The *UBE2A* gene at Xq24 encodes the RAD6 ubiquitin-conjugating enzyme and has been recently shown to be involved in histone modifications that control gene expression [69,70]. The *SMS* gene at Xp22.11 encodes for an enzyme involved in polyamine synthesis and recycling and is directly related to decarboxylated SAM. Previous studies have suggested that alterations in this polyamine synthesis could result in an excess of SAM and may lead to aberrant DNAm status [71].

Taken together, these studies show that DNAm episignatures can be detected in genes with various functions and provide strong evidence for the clinical utility of episignatures as diagnostic biomarkers in NDDs [43,44,72], while also enabling broader understanding of the clinical associations and biological roles of DNAm in genetic disorders.

## 5. Current Episignature Detection in NDDs

We recently described an approach to episignature mapping and development of a clinical EpiSign classifier in 65 genetic syndromes [43] involving bisulfite converted peripheral blood samples analyzed using methylation microarrays. Blood presents itself as the ideal tissue type for episignature development as it is a common clinical sample type and is easily accessible. Since episignatures represent a fundamental defect in NDDs caused by genetic variation in the germline, DNAm changes will be present in all subsequent tissues. This microarray technology enables a genome-wide, cost-effective, standardized, scalable and high throughput assessment of DNAm patterns, amenable to clinical validation in a diagnostic laboratory setting. This technology enables simultaneous assessment of up to 850,000 CpGs across the genome. By applying a custom bioinformatic pipeline to the methylation data obtained from these arrays, we are able to identify sensitive and disorder-specific episignatures. Using unsupervised machine learning models (MLMs), the sensitivity of an episignature can be assessed. Construction of multiclass supervised MLMs to compare a patient’s DNAm data against controls and samples from other clinically validated episignatures at the same time, through the use of an expansive tissue-specific database, should be applied to confirm the specificity of a signature [44]. These methods rely on the ability to perform concurrent assessment of multiple disorders and controls, and highlights the importance of development of large-scale reference databases. To use episignatures in different tissues, a reference database would be required to establish the unique DNAm changes in the particular tissue that arise in development during differentiation and determine how this may impact the specific episignature. Our group has focused on episignatures in blood and has not assessed peripheral blood episignatures in other tissue types. Use of these supervised and unsupervised MLMs is also important when we consider the scalability of testing, as the list of episignatures continues to expand, requiring these algorithms to be capable of handling large amounts of data and computations in a cost-effective and timely fashion.

The ability to detect episignatures is highly contingent upon the intensity (effect size) and extent (number of differentially methylated CpGs) of the observed DNAm changes [44]. Some disorders, such as Sotos or TBRS, are associated with robust changes to the extent of involvement of tens of thousands of CpGs. In contrast, disorders such as the BAFopathies only exhibit a few hundred differentially methylated CpGs [64]. In light of this, sample size can play a role in the ability to detect episignatures in disorders associated with mild or moderate DNAm changes.

When analyzing methylation effects, it is important to consider confounding biological factors such as age, sex and blood cell composition, which are known to be associated with changes in methylation patterns in healthy individuals [73,74]. Methods should be in place to account for such factors when trying to decipher which observed methylation changes contribute to the underlying NDD.

To identify regions containing methylation changes, referred to as differentially methylated regions (DMRs), a ‘bump hunting’ approach [75] can be used, which typically considers regions containing 3–5 CpGs with greater than 10% methylation change between case samples and controls, and gaps of no more than 500 bp between neighboring CpGs [53]. DMRs can be useful in determining significant downstream effects of gene disruption and pathogenesis of disorders such as up- and down-regulated gene expression.

The complex bioinformatic pipeline required to identify episignatures, and to overcome previously mentioned confounding variables, relies heavily on a large-scale tissue-specific reference DNAm database, as well as bioinformatic and clinical genetic expertise [45]. Broadening utility of episignature assessment in the clinical setting involve screening of patients with suspected NDDs, as well as a functional assay for reclassification of VUSs. 

## 6. The Use of Episignatures in the Diagnosis of NDDs

EpiSign is a clinical genome-wide DNAm test that has been available since 2019 that can detect over 60 disorders in more than 80 genes associated with Mendelian disorders through assessment of peripheral blood DNA [43]. A list of the current disorders detectable by EpiSign version 3 are listed in Table 1.

In parallel with screening for episignatures, EpiSign also permits concurrent detection of FRX in males [37] and common imprinting disorders [76], thereby consolidating tests and reducing the need for additional reflexive testing [45]. In addition, its clinical utility in the assessment and reclassification of VUSs in genes with existing episignatures was recently reported in multiple studies [45,72,77,78]. EpiSign is the first and currently the only genome-wide DNAm clinical test offered for screening individuals with NDDs, and can be used as part of the diagnostic work up or for reclassification of VUSs. The reference EpiSign Knowledge Database (EKD) [44] utilized by the EpiSign assay contains thousands of peripheral blood DNAm profiles from both reference controls and NDD. 

The cost of completing a methylation array is comparable to the cost of most CMAs, and the results are highly reproducible. The assay uses peripheral blood, similar to current CMA platforms, allowing streamlined adaption in the clinical setting given the overlap in equipment and laboratory techniques.

The ability to detect episignatures in patients with NDDs has been shown to increase the diagnostic yield, helping to resolve the diagnostic odyssey. A recent study assessing the clinical impact of EpiSign for patients with rare Mendelian disorders demonstrated a 27.6% diagnostic yield among patients with previous ambiguous/inconclusive genetic findings including genetic VUSs [45]. As episignature discovery expands and more disorders are added to the test repertoire, the diagnostic yield is likely to increase significantly, ultimately benefiting patients, their families and the related health care systems. This expansion in episignatures, however, may bring with it challenges, as it is likely to uncover syndromes with overlapping episignatures which may require the implementation of novel computational methods in order to classify these disorders.

## 7. Episignature Development in CNV-Associated Genomic Disorders Provides Insight into Pathological Mechanism

Changes in DNAm profiles, or episignatures, in patients with large CNV defects associated with genomic disorders have not been systematically studied, and it is plausible that large CNVs, much like gene specific variants, may exhibit unique diagnostic methylation signatures in patients with NDDs. 

**Figure 1:**
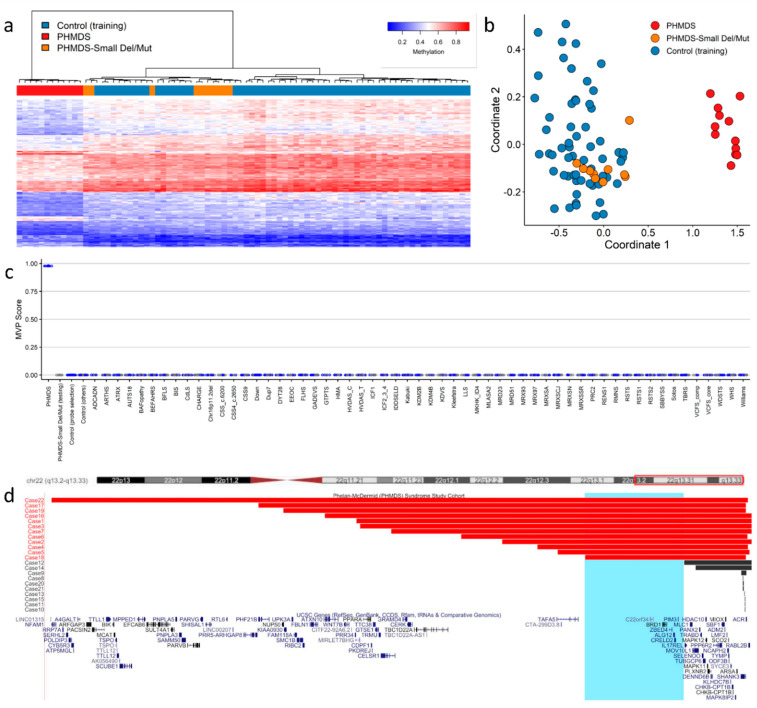
Phelan−McDermid syndrome (PHMDS) episignature demonstrating the critical *BRD1* region: (**a**) Euclidean hierarchical clustering (heatmap); each column represents a single PHMDS case or control, each row represents one of the CpG probes selected for the episignature. This heatmap shows clear separation between large deletion (2−6 Mb in size) PHMDS cases (red) from controls (blue). Smaller deletions (0.01−1 Mb) and intragenic *SHANK3* gene variants (Small Del/Mut) (orange) are shown to segregate with controls. (**b**) Multidimensional scaling (MDS) plot shows segregation of large deletion PHMDS cases from both controls and Small Del/Mut cases. (**c**) Support vector machine (SVM) classifier model. Model was trained using the selected probes for the PHMDS episignature, 75% of controls and 75% of other neurodevelopmental disorder samples (blue). The remaining 25% of controls and 25% of other disorder samples were used for testing (grey). Plot shows the large deletion PHMDS cases with a methylation variant pathogenicity (MVP) score close to 1 compared with all other samples, showing the specificity of the classifier and episignature. (**d**) PHMDS deletions illustrating the critical region of interest associated with DNA methylation episignature. The horizontal red bars represent large deletion PHMDS cases associated with the presence of a distinct episignature. The horizontal black bars represent Small Del/Mut cases that do not have a distinct DNA methylation episignature. Highlighted in light blue is the common critical region of interest (Chr22:49,228,863−50,429,645) of deletions associated with the episignature. The common region of interest contains the candidate *BRD1* gene. Cytogenetic bands and known genes are presented in this figure using the UCSC genome browser [82] 2009 (GRCh37/hg19) genome build. Figure adapted with permission from Schenkel et al. [46].

Our group recently published findings describing episignature discovery in patients with PHMDS [46], highlighting the novel insights DNA methylation analysis can contribute to the pathogenesis of CNV disorders. PHMDS is a genomic disorder associated with deletions of chromosome 22, involving partial or whole-gene disruption of the SH3 and multiple ankyrin repeat domains 3 gene (*SHANK3)*. Intragenic variants in *SHANK3* alone are responsible for a broad range of the phenotypic features observed in PHMDS [79]. However, this gene does not explain the entire phenotype in many patients, particularly speech and motor deficits, as well as renal abnormalities. The phenotypic variability and potential involvement of additional genes within the region has been previously assessed by multiple groups [80,81]. We demonstrated an episignature in patients with large deletions that was not observed in those with small deletions or *SHANK3* gene level variants (Figure 1a–c). The minimal region of difference between these two deletion types, large versus small, included the bromodomain-containing protein 1 gene (*BRD1*), a gene involved in epigenetic mechanisms and a likely candidate gene for the methylation signature observed in these patients (Figure 1d). *BRD1* is a component of a histone acetyltransferase complex that interacts with chromatin remodeling proteins and, before now, there was limited genotype–phenotype association reported in this gene. In addition, metabolic studies confirmed that these patients also exhibited very different metabolic profiles [46], further providing functional evidence for disease pathogenesis, as well as indicating targets for future therapies.

## 8. Defined Episignatures in Other CNV-Associated Genomic Disorders Provide Rationale to Further Expand Episignature Discovery

Symmetrical dose-dependent DNAm profiles have been reported in individuals with deletion of the 7q11.23 region (Williams syndrome; WS) or duplication of the same region (7q11.23 duplication syndrome) [83], highlighting the importance of DNAm in the pathogenesis of these disorders. This region contains a number of genes associated with epigenetic mechanisms, and a study by Aref-Eshghi et al. later showed that these methylation changes resulted in unique episignatures that could differentiate WS and 7q11.23 duplication syndrome from 40 other NDDs and congenital anomaly disorders [44]. In the same study, Aref-Eshghi et al. demonstrated another example of symmetrical DNAm pattern, this time when comparing Hunter–McAlpine syndrome (HMS) and Sotos syndrome. A distinct hypermethylation episignature is observed in HMS patients with duplications involving the 5q35 region containing the *NSD1* gene, a direct contrast to the robust hypomethylation episignature seen in patients with Sotos syndrome, which is the result of loss of function variants in the same *NSD1* gene [44]. 

A DNAm signature was reported in a cohort with the genomic disorder 16p11.2 deletion syndrome (16p11.2DS) [84]—a disorder associated with a variable phenotype that includes increased susceptibility to ASD. Several genes within this region play a role in histone or chromatin function; however, to date, no single candidate gene has been identified to be causative of this disorder or its resultant episignature. Moreover, 16p11.2DS shows reduced penetrance and variable expressivity, and although most deletions are de novo, many are inherited from apparently unaffected parents. These so-called “susceptibility CNVs” present challenges for clinicians in counselling families [41]. Due to the presence of a cluster of LCRs in this region that mediate CNVs through NAHR, there is a reciprocal duplication disorder (16p11.2 duplication syndrome) with similar diagnostic challenges. Studying methylation changes in patients with these susceptibility CNVs and their carrier parents could potentially unlock novel insights into the role of aberrant DNAm in reduced penetrance CNV disorders.

Our group recently described an aberrant DNAm pattern in patients with deletions of 12q24.31 encompassing the known histone modifier gene SET domain-containing protein 1B (*SETD1B*), and demonstrated that patients who harbored point mutations within *SETD1B* shared the same methylation episignature [78]. This study highlights that larger CNVs may exhibit the same methylation affects as gene specific variants within these regions. 

The most common genomic disorder is a 22q11.2 deletion syndrome and is the result of a 1.5–3 Mb deletion also mediated by NAHR at a cluster of LCRs. Clinical manifestations of this disorder include DiGeorge and Velocardiofacial syndromes, and, to date, the phenotype–genotype relationship has not been fully elucidated. Through analysis of a cohort of individuals with 22q11.2 deletions, we identified an episignature that can differentiate 22q11.2 deletion syndrome from other NDDs on the clinical EpiSign test, including those considered in the differential diagnosis of this syndrome [85]. Among other findings, assessment of DMRs showed overlap with loci for orofacial clefting, a key phenotypic feature of this disorder. Through further analysis of atypical deletions and gene level variants, it may be possible to determine the gene, or genes, that play a role in the aberrant DNAm pattern observed, as well as insight into the mechanisms contributing to this disorder. 

Only a few of the most prevalent genomic disorders have a candidate gene considered responsible for the entire phenotypic spectrum. Interestingly, where these candidate genes have been identified, they are predominantly involved in epigenetic regulation including chromatin remodeling or histone modification, e.g., *CREBBP* in Rubinstein–Taybi syndrome [86] and *NSD1* in Sotos syndrome [87] (Table 2). Variants in most of these genes have already been assessed for genome-wide DNAm changes, and have been shown to exhibit unique and specific episignatures [43]. Overall, the majority of CNV disorders do not have a known or suspected candidate gene of interest. However, almost all of these regions contain one or more genes with epigenetic function (Table 2), e.g., chromodomain helicase DNA-binding protein 1-like (*CHD1L*) gene in 1q21.1 deletions and duplications, a gene that has a role in chromatin remodeling following DNA damage [88]. 

Taken together, the evidence suggests that CNV-associated genomic disorders may exhibit aberrant DNAm as the result of genes affected in their underlying deletions and duplications, especially when those regions include genes with epigenetic regulatory roles. CNV-associated genomic disorders are therefore strong candidates for episignature discovery. Investigating these syndromes further, including atypical CNVs and gene level variants within the same regions for possible sub-signatures, may uncover novel insights into the pathogenesis of these disorders. These studies may also identify new candidate genes responsible for some of the phenotypic presentation—should sub-signatures be uncovered for specific deleted or duplicated regions—and potentially unlock novel targets for more personalized treatment approaches.

## 9. Combined Detection of CNVs and DNA Methylation Episignatures in a Single Assay

Recent studies have shown it is possible to detect CNVs by applying computational methods to data obtained from DNAm arrays, such as the Illumina 450K and EPIC Bead Chip arrays [89,90,91]. Many of these pipelines are publicly available in Bioconductor, e.g., ChAMP [91,92], CopyNumber450k [93] and EpiCopy [89] (https://bioconductor.org/packages/, accessed on 19 May 2022). The ability to integrate the detection of genetic and epigenetic findings can provide a more complete view of underlying pathogenic mechanisms. 

We applied a similar computational approach using the DNAcopy package (Bioconductor.org) to our PHMDS cohort, and confirmed we could detect breakpoint coordinates similar to those obtained from conventional clinical CMA at the time of original diagnosis [46]; these findings are in line with previous studies [89,90,91,92,93]. 

Combining these detection methods is not without challenges, most notably in coverage of the genome, as CpG sites are not uniformly distributed throughout the genome and therefore methylation arrays lack the “backbone coverage” observed in high-density SNP arrays. However, it is plausible that, with modifications, a combined array could be developed containing a combination of copy number and CpG targeted probes to produce a clinically targeted array enabling accurate episignature and CNV analysis on a single platform. This has the potential to impact healthcare resource utilization by reducing concurrent testing in NDD patients, and decreasing the need for reflexive testing for disorders such as those associated with imprinting. There would continue to be limitations in the ability to detect low level mosaicism, as seen with existing CNV platforms; however, studies have shown the ability to detect mosaicism from methylation arrays in Kabuki syndrome 1 [94], imprinting disorders [76] and FRX [37].

Additional benefits of a combined testing platform include those to the patient; a combined array would permit screening for more disorders in a single assay, thereby potentially increasing diagnostic yield over that of the current first-tier clinical test (chromosome microarray), and shortening the time spent in the diagnostic odyssey. This approach could concurrently reduce the burden on clinical services and genetic counselling by providing results for CMA, FRX, imprinting and methylation in a single report, leading to a reduction in requisitions and clinic visits. A combined platform would also benefit oncology studies, where limitations in tumor sample availability can often impact research and diagnosis; this would permit the detection of CNVs and methylation status from the same volume of tissue as traditional testing.

## 10. Conclusions

The identification of episignatures in genomic disorders associated with CNVs could facilitate the expansion of screening capabilities for patients with NDDs, improving the diagnostic yield of clinical testing. This work may also provide novel insights into the pathogenesis of genomic disorders and provide targets for future therapies. The ability to combine CNV and episignature detection into a single assay would reduce the overall cost of testing, increase the number of disorders being screened for, and contribute to the reduction in alternative reflexive and concurrent genetic tests ordered. The benefit to patients and families in reducing wait times and increasing screened disorders, as well as reducing clinician and laboratory burden via fewer clinic visits and fewer genetic tests ordered, would have significant impacts on healthcare resource utilization and the costs associated with the diagnosis of NDDs.

## Figures and Tables

**Table 1:** EpiSign v3 assay gene content.

Syndrome	Episignature Abbreviation	Underlying Gene(s) or Region	OMIM
Alpha-thalassemia mental retardation syndrome	ATRX	ATRX	301040
Angelman syndrome	Angelman	UBE3A	105830
Arboleda–Tham syndrome	ARTHS	KAT6A	616268
Autism, susceptibility to, 18	AUTS18	CHD8	615032
Beck–Fahrner syndrome	BEFAHRS	TET3	618798
Beckwith–Wiedemann syndrome	BWS	Chr11p15 (ICR1, KCNQ1OT1, CDKN1C)	130650
Blepharophimosis intellectual disability SMARCA2 syndrome	BIS	SMARCA2	619293
Börjeson–Forssman–Lehmann syndrome	BFLS	PHF6	301900
Cerebellar ataxia, deafness, and narcolepsy, autosomal dominant	ADCADN	DNMT1	604121
CHARGE syndrome	CHARGE	CHD7	214800
Chr16p11.2 deletion syndrome	Chr16p11.2del	Chr16p11.2 deletion	611913
Coffin–Siris syndrome-1, 2 (CSS1,2)	CSS_c.6200	ARID1A; ARID1B	135900; 614607
Coffin–Siris 1–4 (CSS1–4) and Nicolaides–Baraitser syndrome (NCBRS)	BAFopathy	ARID1B; ARID1A; SMARCB1; SMARCA4; SMARCA2	135900; 614607; 614608; 614609; 601358
Coffin–Siris syndrome-4 (CSS4)	CSS_c.2656	SMARCA4	614609
Coffin–Siris syndrome-9 (CSS9)	CSS9	SOX11	615866
Cohen–Gibson syndrome; Weaver syndrome	PRC2	EED; EZH2	617561; 277590
Cornelia de Lange syndromes 1–4	CdLS	NIPBL; SMC1A; SMC3; RAD21	122470; 300590; 610759; 614701
Down syndrome	Down	Chr21 trisomy	190685
Dystonia-28, childhood onset	DYT28	KMT2B	617284
Epileptic encephalopathy, childhood onset	EEOC	CHD2	615369
Floating-Harbour syndrome	FLHS	SRCAP	136140
Fragile X syndrome	FXS	FMR1	300624
Gabriele de Vries syndrome	GADEVS	YY1	617557
Genitopatellar syndrome (see also Ohdo syndrome, SBBYSS variant)	GTPTS	KAT6B	606170
Helsmoortel–Van der Aa syndrome (ADNP syndrome (Central))	HVDAS_C	ADNP	615873
Helsmoortel–Van der Aa syndrome (ADNP syndrome (Terminal))	HVDAS_T	ADNP	615873
Hunter–McAlpine craniosynostosis syndrome	HMA	Chr5q35-qter duplication	601379
Immunodeficiency, centromeric instability, facial anomalies syndrome 1 (ICF1)	ICF_1	DNMT3B	242860
Immunodeficiency, centromeric instability, facial anomalies syndrome 2,3,4 (ICF2,3,4)	ICF_2_3_4	ZBTB24; CDCA7; HELLS	614069; 616910; 616911
Intellectual developmental disorder-65	KDM4B	KDM4B	619320
Intellectual developmental disorder with seizures and language delay	IDDSELD	SETD1B	619000
Intellectual developmental disorder, X-linked 93	MRX93	BRWD3	300659
Intellectual developmental disorder, X-linked 97	MRX97	ZNF711	300803
Intellectual developmental disorder, X-linked, Snyder–Robinson type	MRXSSR	SMS	309583
Intellectual developmental disorder, X-linked, syndromic, Armfield type	MRXSA	FAM50A	300261
Intellectual developmental disorder, X-linked, syndromic, Claes-Jensen type	MRXSCJ	KDM5C	300534
Intellectual developmental disorder, X-linked syndromic, Nascimento-type	MRXSN	UBE2A	300860
Kabuki syndromes 1, 2	Kabuki	KMT2D; KDM6A	147920; 300867
Kagami–Ogatta syndrome	KOS	Chr14q32	608149
KDM2B-related syndrome	KDM2B	KDM2B	unofficial
Kleefstra syndrome 1	Kleefstra	EHMT1	610253
Koolen de Vries syndrome	KDVS	KANSL1	610443
Luscan–Lumish syndrome	LLS	SETD2	616831
Menke–Hennekam syndrome-1, 2	MKHK_ID4	CREBBP; EP300	618332; 618333
Mental retardation, autosomal dominant 23	MRD23	SETD5	615761
Mental retardation, autosomal dominant 51	MRD51	KMT5B	617788
Mental retardation, FRA12A type	DIP2B	DIP2B	136630
Myopathy, lactic acidosis, and sideroblastic anemia-2	MLASA2	YARS2	613561
Ohdo syndrome, SBBYSS variant	SBBYSS	KAT6B	603736
Phelan–McDermid syndrome	PHMDS	Chr22q13.3 deletion	606232
Prader–Willi syndrome	PWS	Chr15q11 (SNRPN, NDN)	176270
Rahman syndrome	RMNS	HIST1H1E	617537
Renpenning syndrome	RENS1	PQBP1	309500
Rubinstein–Taybi syndrome 1	RSTS1	CREBBP	180849
Rubinstein–Taybi syndrome-1, 2	RSTS	CREBBP; EP300	180849; 613684
Rubinstein–Taybi syndrome-2	RSTS2	EP300	613684
Silver–Russell syndrome 1	SRS1	Chr11p15.5	180860
Silver–Russell syndrome 2	SRS2	Chr7p11.2	618905
Sotos syndrome 1	Sotos	NSD1	117550
Tatton–Brown–Rahman syndrome	TBRS	DNMT3A	615879
Temple syndrome	Temple	Chr14q32	616222
Velocardiofacial syndrome	VCFS	Chr22q11.2 deletion	192430
Wiedemann–Steiner syndrome	WDSTS	KMT2A	605130
Williams–Beuren deletion syndrome (Chr7q11.23 deletion syndrome)	Williams	Chr7q11.23 deletion	194050
Williams–Beuren duplication syndrome (Chr7q11.23 duplication syndrome)	Dup7	Chr7q11.23 duplication	609757
Wolf–Hirschhorn syndrome	WHS	Chr4p16.13 deletion	194190

**Table 2:** Common CNV disorders including the candidate genes involved in the clinical phenotype (where applicable), and genes contained within with reported epigenetic machinery roles.

Syndrome	Chromosome Region	Candidate Gene	Genes in Region with Epigenetic Function
1p36 Deletion/Duplication	1p36	-	ICMT, CHD5, TP73, PMRD16, SKI, NOC2L
1q21.1 Deletion/Duplication	1q21.1	-	CHD1L
1q43q44 Deletion	1q43q44	-	HNRNPU, DESI2, ZBTB18, AKT3
2q11.2 Deletion/Duplication	2q11.2	-	KANSL3, ARID5A
2q13 Deletion/Duplication	2q13	-	MIR4435-2HG
2q37 Deletion	2q37	-	HDAC4, D2HGDH, ING5, HDLBP, PASK
3q29 Deletion/Duplication	3q29	-	PAK2, RNF168
4p16.3 Deletion (Wolf–Hirschhorn)/4p16.3 Duplication	4p16.3	NSD2	NSD2, CTBP1, SLBP, CTBP1, PCGF3
5p15 Deletion (Cri du Chat)/5p15 Duplication	5p15	-	ATPSCKMT, MTRR, NSUN2, LPCAT1, BRD9
5q35 Deletion (Sotos)/5q35 Duplication (Hunter–McAlpine)	5q35	NSD1	NSD1, UIMC1
7q11.23 Deletion (Williams–Beuren)/7q11.23 Duplication	7q11.23	-	METTL27, BUD23, BCL7B, BAZ1B
8p23.1 Deletion/Duplication	8p23.1	-	TNKS
9q34 Deletion (Kleefstra)/9q34 Duplication	9q34	EHMT1	EHMT1
10q22.3q23.2 Deletion/Duplication	10q22.3q23.2	-	WAPL, DYDC1, MAT1A
11p11.2 Deletion (Potocki–Shaffer)/11p11.2 Duplication	11p11.2	-	PHF21A, CD82, ALKBH3
11q13.2q13.4 Deletion	11q13.2q13.4	-	KMT5B
15q11.2 Deletion (non-imprinting region)	15q11.2	-	-
15q11q13 Deletion (Prader–Willi/Angelman)/15q11q13 Duplication	15q11q13	-	HERC2
15q13.3 Deletion/Duplication	15q13.3	-	OTUD7A, KLF13
15q24 (BP0-BP1) Deletion/Duplication	15q24	-	-
15q24 (BP2-BP3) Deletion	15q24	-	SIN3A, COMMD4
15q25.2 Deletion	15q25.2	-	HDGFL3, BNC1
16p13.3 Deletion (Rubinstein–Taybi)/16p13.3 Duplication	16p13.3	CREBBP	CREBBP
16p13.11 Deletion/16p13.11 Duplication	16p13.11	-	NDE1
16p11.2 Distal Deletion/Duplication	16p11.2	-	SH2B1
16p11.2 Deletion/Duplication	16p11.2	-	PPP4C, HIRIP3, PAGR1, INO80E
17p13.3 Deletion (Miller–Dieker)/17p13.3 Duplication	17p13.3	-	HIC1, SMYD4, MYO1C
17p11.2 Deletion (Smith–Magenis)/17p11.2 Duplication (Potocki–Lupski)	17p11.2	RAI1	ALKBH5, RAI1, PEMT
17q11.2 Deletion/Duplication	17q11.2	-	SUZ12
17q12 Deletion/Duplication	17q12	-	HNF1B, TADA2A, AATF, PIGW
17q21.31 Deletion (Koolen–de Vries)/17q21.31 Duplication	17q21.31	KANSL1	KANSL1
22q11.2 Tetrasomy/Triplication (Cat eye syndrome)	22q11.2	-	CECR2, ADA2
22q11.2 Deletion (DiGeorge/Velocardiofacial)/22q11.2 Duplication	22q11.2	-	THAP7, TRMT2A, COMT, HIRA
22q11.2 recurrent region distal type I (D-E/F) Deletion/Duplication	22q11.2	-	TOP3B, PPM1F
22q13.3 Deletion (Phelan–McDermid)	22q13.3	SHANK3	BRD1
Xp11.22 Duplication (MRX17)	Xp11.22	-	HUWE1, HSD17B10, SMC1A

## Data Availability

Not applicable.

## Abbreviations

aCGH, array-based comparative genomic hybridization; ADHD, attention deficit hyperactivity disorder; ASD, autism spectrum disorder; CMA, chromosomal microarray analysis; CNV, copy number variant; DD, developmental delay; DNAm, DNA methylation; DNMT, DNA methyltransferases; FRX, fragile X syndrome; HMS, Hunter–McAlpine syndrome; ID, intellectual disability; LCR, low copy repeats; MLM, machine learning models; NAHR, non-allelic homologous recombination; NCBRS, Nicolaides–Baraitser syndromes; NDD, neurodevelopmental disorder; PHMDS, Phelan–McDermid syndrome; SAM, S-adenosylmethionine; SNV, single nucleotide variants; TBRS, Tatton–Brown–Rahman syndrome; VUS, variant of uncertain significance; WES, whole exome sequencing; WGS, whole genome sequencing; WS, Williams syndrome.
